# Delayed convergence between brain network structure and function in rolandic epilepsy

**DOI:** 10.3389/fnhum.2014.00704

**Published:** 2014-09-09

**Authors:** René M. H. Besseling, Jacobus F. A. Jansen, Geke M. Overvliet, Sylvie J. M. van der Kruijs, Saskia C. M. Ebus, Anton J. A. de Louw, Paul A. M. Hofman, Albert P. Aldenkamp, Walter H. Backes

**Affiliations:** ^1^Epilepsy Center KempenhaegheHeeze, Netherlands; ^2^Research School for Mental Health and Neuroscience, Maastricht UniversityMaastricht, Netherlands; ^3^Department of Radiology, Maastricht University Medical CenterMaastricht, Netherlands; ^4^Department of Neurology, Maastricht University Medical CenterMaastricht, Netherlands

**Keywords:** rolandic epilepsy, structural connectivity, functional connectivity, structure-function correlation, brain maturation, graph theory

## Abstract

**Introduction:** Rolandic epilepsy (RE) manifests during a critical phase of brain development, and has been associated with language impairments. Concordant abnormalities in structural and functional connectivity (SC and FC) have been described before. As SC and FC are under mutual influence, the current study investigates abnormalities in the SC-FC synergy in RE.

**Methods:** Twenty-two children with RE (age, mean ± SD: 11.3 ± 2.0 y) and 22 healthy controls (age 10.5 ± 1.6 y) underwent structural, diffusion weighted, and resting-state functional magnetic resonance imaging (MRI) at 3T. The probabilistic anatomical landmarks atlas was used to parcellate the (sub)cortical gray matter. Constrained spherical deconvolution tractography and correlation of time series were used to assess SC and FC, respectively. The SC-FC correlation was assessed as a function of age for the non-zero structural connections over a range of sparsity values (0.01–0.75). A modularity analysis was performed on the mean SC network of the controls to localize potential global effects to subnetworks. SC and FC were also assessed separately using graph analysis.

**Results:** The SC-FC correlation was significantly reduced in children with RE compared to healthy controls, especially for the youngest participants. This effect was most pronounced in a left and a right centro-temporal network, as well as in a medial parietal network. Graph analysis revealed no prominent abnormalities in SC or FC network organization.

**Conclusion:** Since SC and FC converge during normal maturation, our finding of reduced SC-FC correlation illustrates impaired synergy between brain structure and function. More specifically, since this effect was most pronounced in the youngest participants, RE may represent a developmental disorder of delayed brain network maturation. The observed effects seem especially attributable to medial parietal connections, which forms an intermediate between bilateral centro-temporal modules of epileptiform activity, and bear relevance for language function.

## Introduction

Although rolandic epilepsy (RE) is classically considered a benign epilepsy of childhood, over the last decade it has been associated with inattention, impulsivity, and cognitive complaints (Loiseau and Duché, 1989; Massa et al., 2001). The latter notably involves a range of impaired language abilities, such as oromotor deficits, problems in phonological awareness, compromised written language skills, reading disability, and speech sound disorder (Lundberg et al., 2005; Papavasiliou et al., 2005; Clarke et al., 2007; Northcott et al., 2007; Overvliet et al., 2011). These language impairments can clinically be of greater concern than the seizures, especially since they may persist after spontaneous seizure remission, which in RE is typically seen before the age of 16 years (Hommet et al., 2001; Panayiotopoulos et al., 2008; Monjauze et al., 2011).

In neuroimaging, there has recently been a renewed interest in the integration of the motor and the language system. Several studies have demonstrated that the motor system is not only relevant for language in a straightforward way such as coordination of articulatory movement, but is also involved in purely cognitive aspects of language, such as speech comprehension (Pulvermüller et al., 2006). For an overview, we refer to the recent work by Cappa and Pulvermuller and the references therein (Cappa and Pulvermuller, 2012).

In RE, this moto-lingual integration may be a key concept for linking the epileptic focus in the sensorimotor (i.e., rolandic) cortex to the broad range of associated language impairments. Indeed, using functional MRI (fMRI) it has recently been demonstrated that the integration between the motor and the language system is compromised in RE compared to healthy controls (Besseling et al., 2013b,c). Concordant abnormalities in structural connectivity have also recently been described (Besseling et al., 2013a). However, how these functional and structural abnormalities relate to each other remains to be investigated.

It should be noted that RE manifests during a critical and vulnerable phase of brain maturation. The networks that are formed during this period actually are the result of continuous interactions between the developing structural and functional circuits, among other factors (Andersen, 2003). Neuropathological cues, such as epileptiform discharges, may offset normal developmental trajectories and compromise the final adult network organization (Andersen, 2003). In other words, abnormalities in either structural or functional connectivity (SC and FC, respectively) or their interaction may explain behavioral and cognitive problems in children with RE, as well as their persistence in those that are in seizure remission (Hommet et al., 2001; Panayiotopoulos et al., 2008; Monjauze et al., 2011).

The study of the association between SC and FC is an emerging field of research, which is rapidly gaining interest (Sporns, 2013). Several studies have demonstrated that FC is determined (and constrained) by structural white matter connections (Honey et al., 2007, 2009, 2010; Greicius et al., 2009). Vice versa, simulation studies have shown that functional dynamics are spontaneously self-structuring, and in turn may help to sculpt structural connections (Rubinov et al., 2009). It has been suggested that during normal brain development, the SC-FC correlation increases as white matter connections and network dynamics converge under influence of a common optimization process to improve both network efficiency and efficacy (Hagmann et al., 2010; Supekar et al., 2010).

In epilepsy, abnormalities in either SC or FC have extensively been described, as recently reviewed by van Diessen et al. (2013). However, abnormalities in the SC-FC correlation have hardly been investigated, and to our knowledge not yet in the developing child with epilepsy.

In the current work, the SC-FC correlation is investigated as a function of age in children with RE compared to healthy control children. A modularity analysis is employed to investigate whether potential abnormalities can be localized to certain sub-networks, and graph-theoretical measures are employed to check for underlying aberrant SC and/or FC network organization.

## Materials and methods

### Patient selection

Twenty-two children with RE (8 girls; age, mean ± SD: 11.3 ± 2.0 years) were selected at our specialized epilepsy referral center. Selection criteria included centro-temporal spikes on EEG and concordant seizure semiology representing anarthria, hemiclonia involving the face and/or unilateral extremities, or secondarily generalized seizures. In case of poorly observed nocturnal seizures, post-ictal signs of generalized seizure or confirmation of post-ictal hemiparesis was sufficient for inclusion in case of otherwise typical EEG. Further details were described previously (Overvliet et al., 2013).

In addition, 22 healthy controls were enrolled in the study (11 girls, age 10.5 ± 1.6 years). All patients had an IQ >70 and all controls attended regular education, and had neither (a history of) neurological disorders, nor learning problems.

The parents or guardians of all children gave written informed consent for study participation, and the study was approved by the ethical review boards of both participating institutions.

### Magnetic resonance imaging

Conventional structural magnetic resonance imaging (MRI) was applied, as well as diffusion weighted imaging (DWI) and fMRI. Structural imaging included a T1-weigthed sequence. A 3D fast-spoiled gradient echo sequence was used, employing echo time/repetition time/inversion time (TE/TR/TI) 3.8/8.3/1022 ms, and at a resolution of 1 × 1 × 1 mm^3^. The acquisition time was 8 min.

Also high angular resolution diffusion weighted imaging (HARDI) was performed, employing 66 non-colinear gradient directions (Jones et al., 1999), and a diffusion-sensitizing b-value of 1200 s/mm^2^. In addition, a single minimally diffusion-weighted image (b0-scan) was acquired. An echo planar imaging (EPI) sequence was used with TE/TR 72/6600 ms, resolution 2 × 2 × 2 mm^3^, and acquisition time 9 min.

Resting-state functional MRI (rs-fMRI) involved a task-free T2*-weighted blood oxygen level dependent (BOLD) sequence of 195 dynamic image volumes at a TR of 2 s, resulting in an acquisition time of 6.5 min. Further settings included: EPI sequence, TE 35 ms, 2 × 2 mm^2^ in plane resolution, and 4 mm thick axial slices.

### Cortical parcellation

The probabilistic anatomical landmark atlas of gyri, sulci, and basal ganglia was used to parcellate the cortical and subcortical gray matter (Perrot et al., 2009; Varoquaux et al., 2010). This atlas consists of *N* = 137 volumes, each representing a probabilistic map for a certain brain region. To map this atlas to native T1 space, affine registrations were implemented using SPM (version 8). In addition, deterministic node labels were constructed by assigning each voxel to its region of maximum probability.

### Structural connectivity

The diffusion-weighted data were preprocessed and tractography was performed as previously described (Besseling et al., 2012). Briefly, this involved that the diffusion-weighted volumes were registered to the b0-scan to correct for head motion and EPI distortions using affine registrations as implemented in CATNAP (Coregistration, Adjustment, and Tensor-solving: a Nicely Automated Program). CATNAP is based on FSL routines (FMRIB Software Library) and includes correction of the gradient directions for rotations (Landman et al., 2007; Leemans and Jones, 2009).

Next, constrained spherical deconvolution (CSD) was used to estimate voxel-wise fiber orientation distributions (FODs). CSD FODs can represent multiple fiber orientations per voxel, and thus account for partial volume effects such as within-voxel fiber kissing, crossing and bending (Tournier et al., 2007, 2008, 2012). The CSD response function was estimated from the data employing high fractional anisotropy voxels (FA > 0.7). A CSD order of l_max_ = 8 (i.e., 45 spherical harmonics) was used; for details, see Tournier et al. (2009) and Besseling et al. (2012).

Probabilistic tractography was performed employing MRtrix to extrapolate voxel-wise FODs to global (semi)continuous streamlines (Tournier et al., 2012). Per subject, 50.000 streamlines were seeded from the gray matter (FSL based tissue segmentation of the T1 scan), and propagated over the brain to represent the overall topology of the global white matter network. Standard MRtrix tractography settings were used, which includes a streamline propagation step size of 0.2 mm, a minimum radius of curvature of 1 mm, and an FOD amplitude threshold >0.1.

Structural connectivity (SC) was investigated for the deterministic node labels. For this, the streamlines were first mapped to the T1-space based on an affine registration of the b0-scan to the T1-scan using FSL (Pannek et al., 2011). Next, for each pair of nodes the interconnecting streamlines were assessed. As larger nodes typically contain more streamlines, connection strength was quantified as the number of streamlines normalized for the number of voxels per node pair (van den Heuvel and Sporns, 2011; Zhang et al., 2011).

### Functional connectivity

Preprocessing of the rs-fMRI data included registration of all fMRI volumes to the first dynamic to correct for head motion employing SPM8. Subsequently, the mean rs-fMRI image volume was calculated and used to affinely register the rs-fMRI data to the native T1-space.

The T1 tissue segmentation was downsampled to the rs-fMRI resolution to calculate averaged time series for the white matter and the CSF. These time series, combined with the movement parameters of the previous step, were used as nuisance regressors to deconfound the rs-fMRI data employing linear regression. This procedure is assumed to provide a more specific and robust correction for non-neuronal signal fluctuations such as scanner drift or physiological noise (cardioballistics and breathing) than whole-brain signal regression (Smith et al., 2011).

Finally, the rs-fMRI data were smoothed using a Gaussian kernel of full-width-at-half-maximum 10 mm, and band-pass filtered to confine the signal to the range of 0.01–0.1 Hz as typically used in rs-fMRI analyses (Zalesky et al., 2010; Cocchi et al., 2012; Hong et al., 2013).

Functional connectivity (FC) was assessed using correlation of node pair time series. The probabilistic atlas maps were used to calculate these node time series as a weighted average, effectively assigning more weight to the core and less weight to the border of each node.

### Correlation between structural and functional connectivity

To assess the SC-FC correlation, the FC values were resampled to a normal distribution of mean 0.5 and SD 0.1 following previous work (Honey et al., 2009; Zhang et al., 2011). Furthermore, negative FC values were set to 0 (Smith et al., 2011).

For each subject, the SC-FC association was calculated by appending the SC and FC values of the non-zero structural connections in two separate vectors, which were subsequently correlated. To assess the robustness of the above analysis, it was applied over a range of sparsity values. This means that not all structural connections were included in the correlation analysis, but only a certain top percentage.

**Figure 1:**
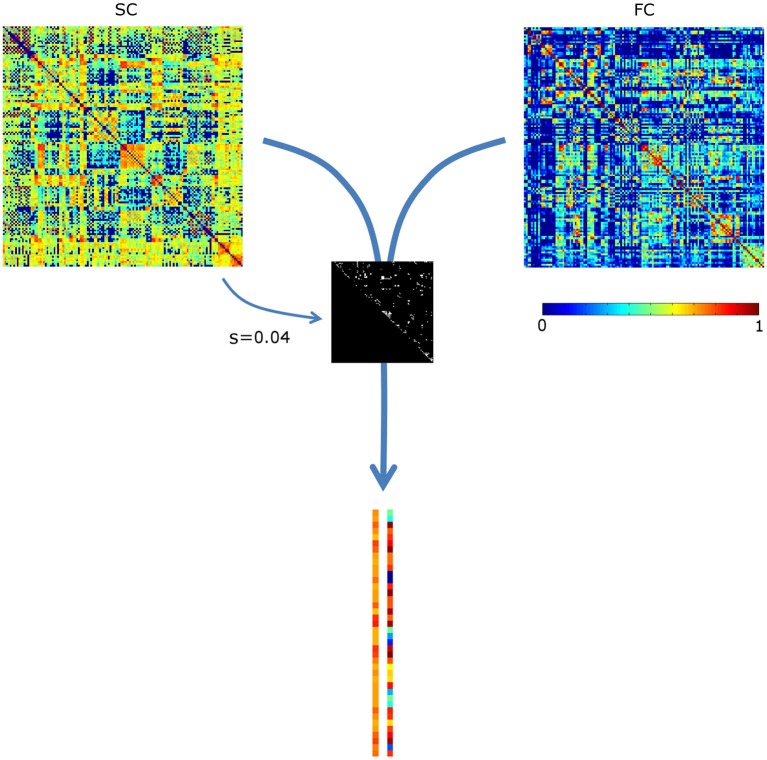
**Assessing the correlation between structural and functional connectivity (SC and FC, respectively)**. The most prominent structural connections (here at sparsity *s* = 0.04) are selected. The SC and FC values of these are appended in two vectors, which are subsequently correlated. For visualization purposes, the vectors are not visualized in full length. SC values were scaled to a Gaussian distribution (mean, SD: 0.5, 0.1); negative FC values were set to 0.

The sparsity structure (i.e., which connections were included) was based on the mean SC matrix of the controls, see Figure 1. Since the mean sparsity of the SC of the controls was 0.75, a sparsity range of 0.01–0.75 was chosen, corresponding to 93–6987 connections. Sparsity values were incremented at a step size of 0.01.

### Modularity analysis

To investigate whether potential abnormalities in SC-FC correlation may be contributed to specific brain sub-networks, a modularity analysis was applied to the average SC network of the controls using the Brain Connectivity Toolbox (BCT). The BCT is a well-established collection of mathematical routines for graph theoretical analysis of complex brain networks (Rubinov and Sporns, 2010). Modularity analysis clusters nodes into modules, based on relatively high within-module and low between-module connectivity (Newman, 2006). Because of heuristics in the algorithm, the modularity analysis was repeated 100 times, and the most robust modularity structure (of highest occurrence) was retained.

### Assessing differences in structure-function correlation

Abnormalities in SC-FC correlation in patients compared to controls were inferred upon using a general linear model (GLM), in which also the effect of age was incorporated. Group differences with respect to SC-FC correlation were deemed significant on the whole-brain level for *p* < 0.05. If such a significant group difference was found, it was investigated whether this could be attributed to one (or multiple) of the sub-networks, employing Bonferroni multiple comparisons correction for the number of sub-networks under investigation.

### Measures of network organization

To investigate whether SC and/or FC as such were abnormal, graph theoretical measures of network integrity were calculated. For each subject, structural and functional network organization were quantified by average path length and clustering coefficient (CC) as assessed using the BCT. Individual SC and FC connectivity matrices were scaled with respect to their total connectivity weight to normalize the “wiring cost” over subjects (Zhang et al., 2011). Potential differences in graph measures thus only reflect differences in the distribution of connectivity values. This is similar to the SC-FC correlation analysis, which also only takes the distribution of the connectivity values into account and not their amplitude. Furthermore, for each subject the graph measures were normalized with respect to comparable random graphs (*N* = 20) (van den Heuvel and Sporns, 2011). The comparisons between patients and controls were performed using the same GLM as described above.

## Results

**Figure 2:**
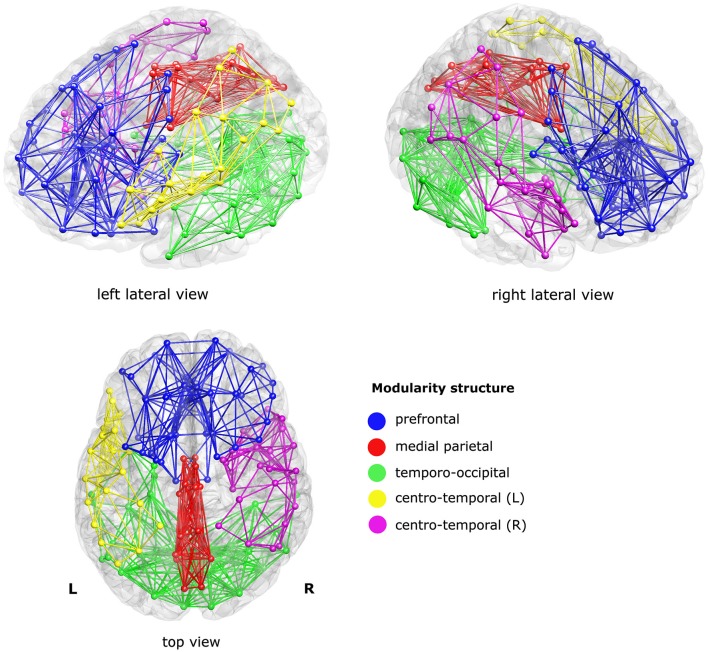
**Sub-networks as derived from a modularity analysis of the mean structural connectivity (SC) network of the controls**. Five sub-networks (i.e., modules) were found; a prefrontal cluster (blue), a medial parietal cluster (red), an inferior temporo-occipital cluster (green), and a left and a right centro-temporal cluster (yellow and magenta, respectively). For visualization purposes, only the within-module connections were visualized, at sparsity level 0.1.

The modularity analysis revealed five clusters: a prefrontal cluster, a medial parietal cluster, an inferior temporo-occipital cluster, and left and right centro-temporal clusters, see Figure 2.

**Figure 3:**
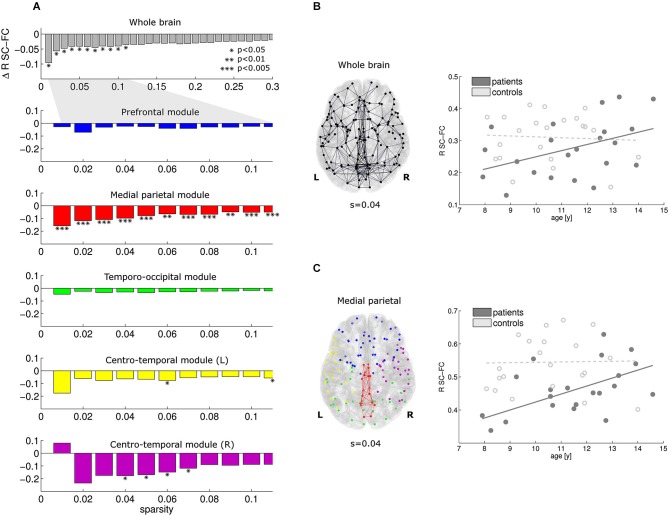
**At the whole-brain level, the correlation between structural and functional connectivity is reduced in patients vs. controls for the sparsity range 0.01–0.11 (A, top)**. Within this sparsity range, similar effects were found in all modules; effects of highest significance were found within the medial parietal module (**A**, module 2). For sparsity value 0.04, the connections investigated and the regression lines are given in **(B)** and **(C)** for the whole brain and the medial parietal module, respectively. Note that the reduction is SC-FC correlation is more pronounced in the medial parietal module. Furthermore, significant increases in SC-FC correlation with age were found in the patients only (solid lines).

At the whole-brain level, significant reductions in SC-FC correlation were found in the patients for the most prominent brain connections (sparsity range 0.01–0.11; Figure 3A, top row). Within this sparsity range, the SC-FC correlation was also consistently reduced within all five modules. Significant effects were found in the medial parietal cluster, and also in both centro-temporal clusters. Within the medial parietal cluster, the reduction in SC-FC correlation was most robust over the sparsity range, and also most pronounced (Figure 3A; *p* < 0.005).

For a representative sparsity value of 0.04, the connections under investigation and the regression lines are given for the whole brain and the medial parietal cluster in Figures 3B,C, respectively. Note the significant progressive increase in SC-FC correlation with age in the patients; no such effect was found in the controls. The group differences were strongest for the youngest participants and diminished towards the end of the age window under investigation (8–14 years).

**Figure 4:**
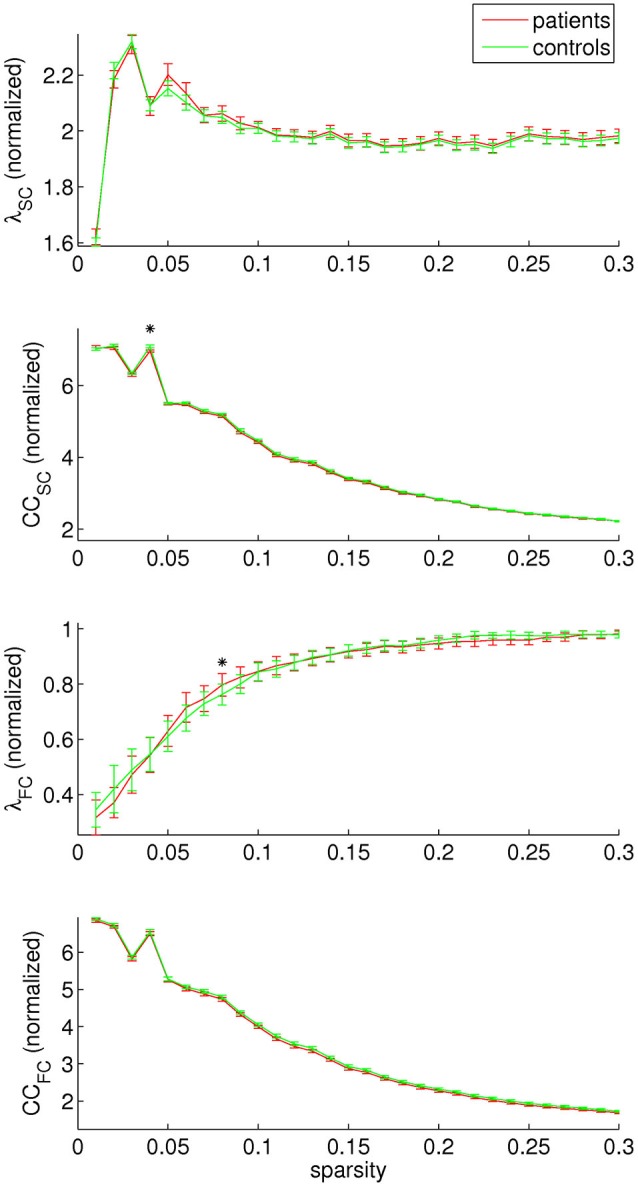
**Structural and functional connectivity (SC and FC, respectively) were investigated separately by assessing the normalized path length (λ) and clustering coefficient (CC)**. A decrease in SC clustering coefficient was found for sparsity value 0.04, and an increase in FC path length for sparsity value 0.08.

Concerning graph analysis, no clear differences in either SC or FC network organization were found between patients and controls. For single sparsity values, a decrease in SC clustering coefficient and an increase in FC path length were found (sparsity 0.04 and 0.08, respectively), see Figure 4. No consistent effects were found over any of the modules under investigation.

## Discussion

In this study, we investigated the correlation between SC and FC as a function of age in children with RE compared to healthy controls. We employed a modularity analysis to investigate whether potential effects on the whole-brain level may be attributed to certain sub-networks. Additionally, graph theoretical measures of network organization were used to assess SC and FC separately, both at the global level and concerning the identified modules.

### Major findings

Our main results are:

1. In children with RE, the SC-FC correlation is reduced compared to healthy controls at the whole-brain level;
2. The SC-FC correlation increases with age in the children with RE, implying that the reduction in SC-FC correlation is less pronounced towards the end of the age window under investigation (8–14 years);
3. Similar effects were found at the module level, i.e., for both centro-temporal clusters and most notably for the medial parietal cluster, in which the reduction in SC-FC correlation was stronger than at the whole-brain level;
4. Employing graph analysis, no prominent abnormalities in SC or FC network organization were found.

### Interpretation of differences in SC-FC correlation

During normal brain maturation, network structure and function gradually adapt to each other, leading to an increase in the SC-FC correlation (Hagmann et al., 2010; Supekar et al., 2010). In adults with idiopathic generalized epilepsy, reductions in SC-FC correlation have been described which are more pronounced for increased disease duration (Zhang et al., 2011). From these findings, one may deduce that an increase in SC-FC correlation signifies an improvement of network organization, and vice versa. In contrast, increased SC-FC correlation has recently been reported in idiopathic generalized epilepsy as well (Liao et al., 2013).

To understand these seemingly contradictory results, note that the correlation between SC and FC only quantifies how well brain structure and function match, but conveys no direct information on structural or functional network integrity as such. During normal brain development, SC and FC may converge driven by a common optimization process to improve network efficiency and efficacy (Andersen, 2003). In epilepsy, on the other hand, various scenarios may apply. If brain structure and function are differentially affected by the epilepsy, SC and FC may diverge and the SC-FC correlation decreases. However, if both SC and FC strongly reflect epileptic remodeling, this may actually increase the SC-FC correlation. To which extent either of these scenarios applies depends on the type of epilepsy, the disease duration, the seizure frequency, and anti-epileptic drug (AED) use, among other factors (van Diessen et al., 2013).

### Measures of network organization

To interpret the current finding of reduced SC-FC correlation in children with RE compared to healthy controls, we assessed potential abnormalities in structural and functional network integrity separately employing graph theoretical measures. A decrease in SC clustering coefficient and an increase in FC path length were found. Both findings may represent reduced network integrity, and similar findings have been reported in epilepsy before (van Diessen et al., 2013). However, although these effects were within the sparsity range of global SC-FC correlation effect (0.01–0.11), they were only found for single sparsity values, and within this sparsity range, no module effects were found. These findings suggest that in RE, gross abnormalities in structural and/or functional network organization are not the case. Indeed, RE is a relatively mild disorder compared to other epilepsy syndromes within the same spectrum, such as Landau Kleffner Syndrome (Hughes, 2010; Overvliet et al., 2010). Our finding of reduced SC-FC correlation should thereby be interpreted in the context of aberrant brain maturation, and reflects a reduced convergence between network structure and function. In agreement, cortical abnormalities, reflective of impaired development of the underlying network, have been reported in RE before (Overvliet et al., 2013).

### Effect of age

Note that the SC-FC correlation actually progressively increased with age in the patients, whereas no age effect was found in the controls. This suggests that the maturational convergence of SC and FC is merely delayed in RE. Potentially, for the age range under investigation (8–14 years), network structure and function are already to a certain extent matched for the controls, whereas in the children with RE, this optimization process is still ongoing. However, since it has been reported before that the cognitive complaints may persist, a full recovery of the SC-FC correlation is not expected (Hommet et al., 2001; Andersen, 2003; Monjauze et al., 2011).

### Localization to sub-networks

Concerning the localization of effects, the overall reduction in SC-FC correlation seems to be particularly attributable to a medial parietal network, where the effect was actually more pronounced than at the whole-brain level. Note that the parietal lobe has been associated with visuo-spatial skills, which may be impaired in RE (Pinton et al., 2006; Völkl-Kernstock et al., 2006).

More importantly, the medial parietal module provides an interface between the bilateral centro-temporal modules, from which the epileptiform activity originates (i.e., RE typical centro-temporal spikes). Spread of epileptiform activity to parietal regions has been demonstrated in RE before (Drury and Beydoun, 1991; Graf and Lischka, 1998). Furthermore, the SC-FC correlation appeared (to a lesser extent) compromised within these centro-temporal modules themselves as well. Abnormalities in the left centro-temporal module may translate to the prominent language impairments in RE, as this module roughly covers classical inferior frontal and supramarginal language areas (Broca’s and Wernicke’s, respectively). Furthermore, since language lateralization is limited in children in general (Kadis et al., 2011) and in RE in particular (Besseling et al., 2013c), impairments of contralateral (right hemisphere) homotopic cortex may also relate to compromised language skills.

The module effect was more robust for the medial parietal module than e.g., for the left centro-temporal module, which might be due to variations in the laterality of EEG abnormalities. Defining a homogeneous RE cohort with respect to electrophysiology is very challenging as the laterality (and extent) of the EEG abnormalities may dramatically change over time (Riva et al., 2007). Actually this is considered a general confound for epilepsy connectivity studies (van Diessen et al., 2013). Since in our clinical cohort electrophysiology was assessed during the diagnostic work-up and not at the time of scanning, the patients can be assumed to be somewhat heterogenous with respect to EEG characteristics. On the group level, an un-lateralized module effect may therefore be the most robust.

### Methodological consideration and directions for future research

Concerning the localization of the network impairments, it is unclear whether the modularity structure employed in this study was optimal to detect aberrant sub-networks in RE. The sub-networks we investigated were derived from an unbiased graph theoretical modularity analysis of the structural data. For the atlas we employed, this yielded a robust set of five compact clusters, two of which were clearly lateralized. These robust clusters had a relatively straightforward anatomical description and interpretation.

Note that specific investigations of e.g., the language network may have yielded more illustrative results in the context of the RE-typical neuropsychological profile of language impairment (Overvliet et al., 2010). However, this would be at the cost of subjective choices, since at present the language network cannot unambiguously be identified from e.g., functional connectivity patterns (Beckmann et al., 2005; Besseling et al., 2013c).

In any case, our findings illustrate that investigations into SC-FC correlation can be used to infer where in the brain neuronal network formation is impaired, and as such our study extends the work by Zhang et al. on generalized epilepsy to localization-related epilepsies such as RE (Zhang et al., 2011).

In our cross-sectional study, the effect of age could only be assessed by virtue of the variability in age of the subjects included. Longitudinal studies are called for to verify the robustness of our findings. Follow-up until after seizure remission is proposed for to investigate to what extent the reported abnormalities may eventually normalize.

Finally, for practical reasons AED use was not controlled for in the clinical cohort under investigation. As described previously, about two thirds of the patients were under AED treatment, which may have confounded our results (Overvliet et al., 2013). The effects described are expected to be more pronounced for a more tightly controlled patient group.

## Conclusion

In children with RE, the correlation between SC and FC is reduced compared to healthy controls. As structural nor functional network organization is much affected, this is interpreted as reduced synergy of SC and FC due to aberrant brain maturation. Since in the patients the discrepancy between SC and FC improves as a function of age, these findings may actually represent maturational delay. Concerning the localization of brain network abnormalities, the observed effects seem especially attributable to medial parietal connections, which forms an intermediate between bilateral centro-temporal modules of epileptiform activity.

## Conflict of interest statement

The authors declare that the research was conducted in the absence of any commercial or financial relationships that could be construed as a potential conflict of interest.
